# Kikuchi-Fujimoto disease evolves into lupus encephalopathy characterized by venous sinus thrombosis: a case report

**DOI:** 10.3389/fimmu.2024.1389993

**Published:** 2024-04-11

**Authors:** Wenyi Qin, Shuangshuang Yang, Lijuan Zhang, Mengqi Liu, Jiayu Tian, Juan Yang, Guoqing Zhou, Xiaofeng Rong

**Affiliations:** ^1^ Department of Rheumatology and Immunology/Department of Integrated Traditional Chinese and Western Medicine. The First Affiliated Hospital of Chongqing Medical University, Chongqing, China; ^2^ Department of Laboratory, The First Affiliated Hospital of Chongqing Medical University, Chongqing, China; ^3^ Department of Radiology, The First Affiliated Hospital of Chongqing Medical University, Chongqing, China

**Keywords:** neuropsychiatric lupus, venous sinus thrombotic, Kikuchi-Fujimoto disease, necrotizing lymphadenitis, cerebrovascular disease

## Abstract

Kikuchi-Fujimoto disease (KFD) is a benign, self-limiting illness that can progress to systemic lupus erythematosus (SLE) in approximately 30% of cases. Neurological injuries can occur in both diseases, albeit with distinct presentations. Venous sinus thrombosis is a serious cerebrovascular complication in patients with neuropsychiatric SLE but is rarely observed in patients with KFD. The involvement of various antibodies, particularly antiphospholipid antibodies, can cause vascular endothelial cell injury, resulting in focal cerebral ischemia and intracranial vascular embolism in SLE. However, there are cases in which thrombotic pathology occurs without antiphospholipid antibody positivity, attributed to vascular lesions. In this report, we present a case of KFD and lupus encephalopathy featuring cerebral venous sinus thrombosis, despite the patient being negative for antiphospholipid antibody. We also conducted a comparative analysis of C3 and C4 levels in cerebrospinal fluid (CSF) and peripheral blood, along with the protein ratio in CSF and serum, to elucidate the pathological changes and characteristics of lupus encephalopathy.

## 1 Introduction

Kikuchi-Fujimoto disease (KFD) is characterized by lymph node enlargement, featuring subacute necrotizing lymphadenopathy with symptoms such as swollen lymph nodes, pain, and fever. The lymph node biopsy reveals a proliferative and necrotizing process centered in the paracortex, characterized by patchy circumscribed or confluent areas of necrosis associated with karyorrhexis and marked by the absence of granulocytes and the paucity of plasma cells (1). These distinctive features could differentiate KFD from nearly all other forms of nodal hyperplasia. Biopsy is crucial for KFD diagnosis, which is linked to various infectious and autoimmune diseases, including systemic lupus erythematosus (SLE) (2). While KFD is sometimes seen as a precursor to SLE (3, 4), its direct impact on SLE activity or prognosis remains unclear. Even in severe cases involving neurological damage, distinguishing KFD-related damage from SLE-induced lupus encephalopathy requires serum immunology and imaging examinations.

Neuropsychiatric SLE (NPSLE) is a severe complication involving the nervous system (5), displaying diverse manifestations ranging in severity with prognostic implications. NPSLE includes various neurological and psychiatric symptoms, often challenging to differentiate from unrelated events. Common complaints in SLE patients encompass headaches, mood disorders, anxiety, and mild cognitive dysfunction, which may not reflect disease activity in the central nervous system (CNS) and can be misdiagnosed or overlooked (6). Cerebral venous sinus thrombosis (CVST) is a rare vascular lesion within NPSLE, occurring even in patients without antiphospholipid antibodies, typically presenting with intracranial hypertension and headaches (7). Diagnostic tools such as cerebral magnetic resonance venography (MRV) or computed tomography venography (CTV) are crucial for diagnosing NPSLE with CVST. Active SLE control coupled with anticoagulant therapy improves NPSLE prognosis, and identifying assessment methods and biomarkers for NPSLE is vital to avoid misdiagnosis.

This report presents a unique case of a patient with SLE initially diagnosed with KFD, later developing CVST during immunosuppressive treatment. Headache and anxiety masked the true condition, and thorough serum immunological tests and imaging exams were crucial for diagnosis and treatment, leading to positive therapeutic outcomes. This case contributes to a deeper understanding of the characteristics and mechanisms of NPSLE.

## 2 Case presentation

**Figure 1:**
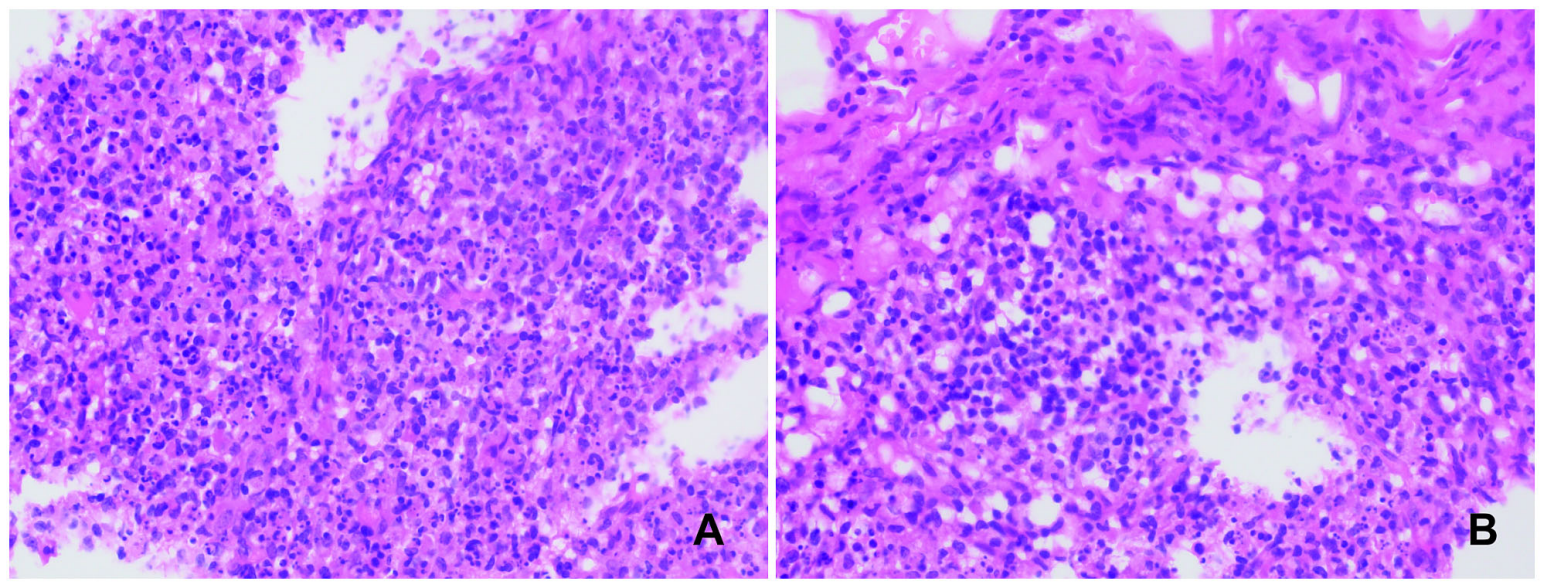
Left subclavian lymph node biopsy. Conventional hematoxylin and eosin staining (H&E), 400X. Multiple lesions containing a significant number of histiocytes and nuclear debris are observed in the lymph nodes. No neutrophils or plasma cells are seen **(A, B)**.

A 25-year-old woman was admitted to our hospital on November 11, 2023, with a high fever (peaking at 39°C) and lymph node pain persisting for 1 month. We performed a lymph node pathological biopsy (**Figures 1A, B**), which led to the diagnosis of KFD (necrotic lymphadenitis). Treatment with anti-inflammatory medication (40 mg methylprednisolone PO qd) led to normalization of her body temperature and significant alleviation of neck lymph node pain. However, during the course of her illness, she developed increased anxiety and headaches, likely due to work stress. After consultation with a psychiatrist, she was diagnosed with a mild anxiety state, but treatment with alprazolam did not significantly improve her symptoms. Due to concerns about potential neurological complications from KFD, a cerebral MRI scan was conducted, which raised suspicion of subarachnoid hemorrhage or infectious lesions. Subsequently, a lumbar puncture and CSF examination were performed, revealing normal results (routine, biochemical, and smear), with Cl^−^ at 121 (120–132mmol/L), Glu at 3.1 (2.2–3.9mmol/L), Pro at 0.23 (0.12–0.60g/L), and negative findings for acid bacteria, *Cryptococcus* fungi, and bacteria, with slightly elevated CSF pressure (230 mmH_2_O; normal: 80–180 mmH_2_O).

**Figure 2:**
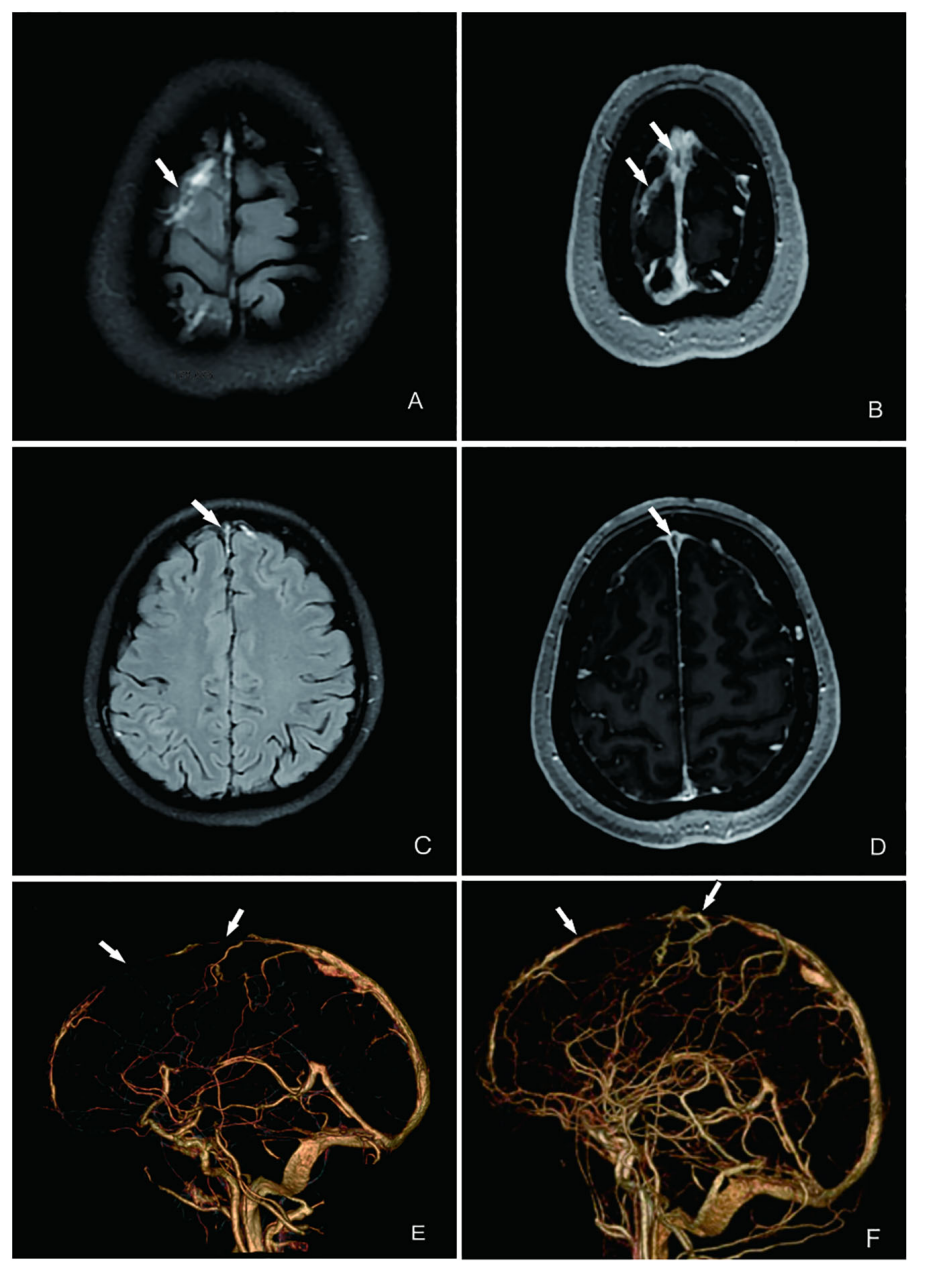
MRI and CTV of the patient’s brain showing multiple intracranial thrombosis in the venous sinus. **(A)** T2 imaging suggests multiple superficial venous thrombosis (indicated by the arrow). **(B)** T1-enhanced imaging showing the same results as those shown in **(A)**. **(C)** T2 flare imaging indicating superior sagittal sinus thrombosis (indicated by the arrow). **(D)** T2-enhanced imaging showing the same results as those shown in **(C)**. **(E)** CTV showing a filling defect in the superior sagittal sinus on CTV, indicating thrombosis formation. **(F)** After treatment of CTV, partial resolution of the superior sagittal sinus thrombus and improvement in thrombus severity are seen, compared with before treatment.

The frequent headaches and anxiety symptoms observed did not align with the typical manifestations of KFD, such as aseptic meningitis (7). Considering the comprehensive clinical manifestations, sex, age, and other factors, we suspected an alternative diagnosis despite the initial KFD diagnosis. An immunological examination was conducted (**Table 1**), revealing a definitive diagnosis of SLE. Both the headache and anxiety were attributed to SLE, leading to the initiation of treatment with methylprednisolone (80 mg IVGTT D1-D7), mycophenolate mofetil (750 mg bid PO), and hydroxychloroquine sulfate (200 mg bid PO). However, after 7 days of therapy, there was no significant improvement in her headache and anxiety symptoms, prompting further investigation. Cerebral MRA+MRV (**Figures 2A–D**) and CTV (**Figure 2E**) scans revealed superior sagittal sinus thrombosis as the cause of her headache. An electroencephalogram also indicated mild abnormalities, confirming the presence of NPSLE.

**Table 1:** Antibodies, immunological test results, and reference interval.

Antibodies	Results	Reference interval
ANA	**1:1000 granular type**	Negative
Anti-U1-nRNP	Negative	Negative
Anti-Smith	Negative	Negative
Anti-SSA	**Positive**	Negative
Anti-Ro52	**Positive**	Negative
Anti-dsDNA	Negative	Negative
Ribosomal P protein	Negative	Negative
Complement 3	**0.60**	0.79–1.52g/L
Complement 4	0.16	0.16–0.38g/L
IgG	**17.80**	7.51–15.60 g/L
IgM	0.71	0.46–3.04 g/L
IgA	1.99	0.82–4.53 g/L
Anti-β2 GP1	Negative	Negative
Antiphospholipid	Negative	Negative
LA1/LA2	1.09	<1.20
Anti-dsDNA	9.9	0–100 IU/mL
ANCA	Negative	Negative

ANA, Antinuclear antibodies; ANCA, antineutrophil cytoplasmic antibodies; anti-β2-GP1, anti-beta-2 glycoprotein 1; dsDNA, double-stranded DNA; Ig, immunoglobulin; LA, lupus anticoagulant; RNP, ribonucleoprotein.

Bold font prompted abnormal results.

The final diagnosis for the patient was identified as NPSLE secondary to KFD, characterized by CVST. To assess blood–brain barrier (BBB) permeability and understand the pathological mechanism of NPSLE, the albumin CSF-to-serum ratio was calculated, revealing an albumin CSF concentration of 0.23 g/L, albumin serum concentration of 42 g/L (normal: 40–55g/L), and albumin CSF/serum ratio exceeding 5.48×10^−3^.

**Figure 3:**
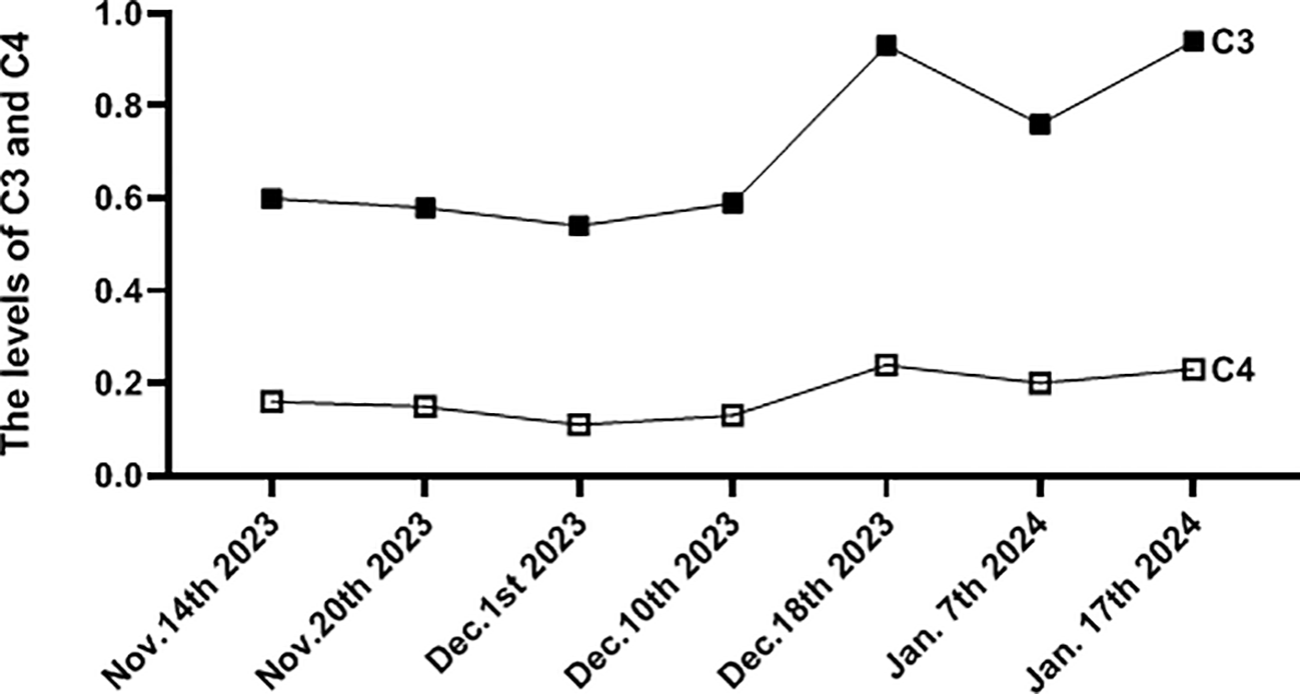
The picture showed the levels change of C3 and C4 during the process of treatment. (g/L, reference value C3,0.79-1.52; C4, 0.16-0.38).

Treatment adjustments included transitioning to intravenous infusion of cyclophosphamide at a dosage of 600 mg over 2 days, tapering methylprednisolone dosage to 50 mg per day orally, and administering hydroxychloroquine sulfate (HCQ) at 200 mg twice daily orally. Importantly, low-molecular-weight heparin was administered for its antithrombotic effects. After 4 weeks of treatment, significant alleviation of headache and anxiety was observed, along with significant improvement in the patient’s overall well-being. A repeat cerebral CTV was scheduled to reassess the status of CVST, which revealed marked reduction in venous sinus thrombosis and recanalization of the thrombus (**Figure 2F**), along with significantly increased activity markers of C3 and C4 (**Table 1**, **Figure 3**). The patient is currently under close monitoring with regular follow-up appointments.

## 3 Discussion

NPSLE is a severe complication of SLE that affects the nervous system, manifesting as neurological or psychiatric symptoms. The underlying mechanisms of NPSLE are complex and may involve genetic factors, dysfunction of the BBB, vascular lesions, autoimmune antibodies, cytokines, and neuronal cell death, all potentially contributing to NPSLE development (5, 8). Autopsy findings in patients with NPSLE suggest that damage to the CNS is linked to cerebrovascular lesions, with common pathological manifestations including cerebral microvascular ischemia and thrombosis, small vessel non-inflammatory lesions, focal vascular occlusion, and microhemorrhage. Several studies have indicated that antiphospholipid (aPL) antibodies are associated with diffuse NPSLE syndromes, indicating a broader pathogenic role beyond their prothrombotic effects (9).

CVST is rare in patients with SLE, with a low incidence of approximately 0.36% (10). However, in this case, the patient experienced multiple CVST without positive aPL antibodies. We hypothesize that cerebrovascular disease in such patients is primarily related to immune inflammation, immune complex deposition, and activation of the complement pathway. Factors such as defective fibrinolysis, altered antithrombin III function, hyperfibrinogenemia, or coagulation changes may also play a role (11, 12). The presence of multiple thromboses in the cerebral venous system suggests a hypercoagulable state in the patient’s body. Despite the absence of aPL antibodies, the high SLE activity and severity of the injury were evident from the neurological damage and SLEDAI score. Treatment with immunosuppressants and anticoagulants proved effective, indicating that immune complex-induced vasculitis may play a significant role in CVST pathogenesis.

Additionally, this case initially diagnosed as KFD due to neurological symptoms and positive auto-antibodies highlights the importance of distinguishing between neurological damage caused by KFD and NPSLE. As KFD shares some clinical and histological features with SLE, it has been proposed that KFD may represent a self-limiting SLE-like autoimmune disorder (13, 14). CNS involvement, such as aseptic meningitis, meningoencephalitis, acute cerebellar symptoms with tremors and ataxia, and optic neuritis, has been reported in KFD (15, 16), with aseptic meningitis being a common complication. However, the mechanisms of deep vein thrombosis and disseminated intravascular coagulation occurring within KFD are rarely reported. For this case, CVST without positive aPL antibodies may involve endothelial damage, enhanced expression and release of procoagulant substances by granulocytes and macrophages, and activation of the coagulation cascade (17, 18). If symptoms persist or new ones arise after treatment, other diseases should be considered. Severe headache and anxiety should alert clinicians to identify the actual causative factor. For young women with underlying KFD, the possibility of autoimmune diseases such as SLE should be considered if presenting symptoms persist despite standard treatment. Auto-antibody testing can aid in diagnosis. Atypical symptoms such as autoimmune hemolytic anemia and the need for rheumatology referral and follow-up for possible development of SLE are crucial aspects to consider (19). Moreover, investigating the cytokines and inflammatory markers associated with KFD could elucidate the thrombotic mechanisms.

The overactivation of the complement pathway can lead to neuronal damage in various neurological diseases (20). We assessed C3 and C4 levels in peripheral blood and CSF to elucidate the function of the BBB in NPSLE. Several studies on immune-mediated diseases have measured CSF concentrations of C3 and C4 to evaluate the role of the complement system. However, measuring C3 and C4 concentrations in CSF alone is insufficient for detecting intrathecal complement production in the CNS. Despite complement components being locally produced by resident brain cells (21), their synthesis in the brain is low or undetectable under healthy conditions (22). Consequently, BBB damage permits complement entry from the systemic circulation, resulting in elevated complement concentrations in CSF. Our case results revealed negative detection of C3 and C4 levels in CSF, which significantly differed from those in peripheral blood, although they were markedly decreased in serum. Comparing complement C3 and C4 levels in the CSF of patients with lupus encephalopathy and non-SLE patients showed no marked differences, indicating that diagnosing and assessing NPSLE based on CSF and serum C3 and C4 levels alone is inadequate. Several reports provide methods to evaluate the function of the BBB and NPSLE activity. The albumin CSF-to-serum ratio serves as a surrogate marker for BBB permeability (23) and can help assess CNS inflammation. In this case, the elevated albumin CSF-to-serum ratio of 5.48×10^−3^, exceeding the normal value of 5.0×10^−3^, indicated BBB injury in the patient, highlighting the role of immune inflammation in BBB permeability.

## 4 Conclusion

This case report emphasizes the differences between KFD anNPSLE in terms of neurological injury and management. CVST in NPSLE can occur without positive aPL antibodies and requires distinct treatments compared to KFD. NPSLE treatment depends on disease activity and organ damage severity. Anticoagulation and antiplatelet therapies are essential for CVST cases. Additionally, assessing changes in C3 and C4 levels in CSF alone is inadequate for evaluating NPSLE activity, but the CSF-to-serum protein ratio can effectively demonstrate BBB injury. Further research is needed to understand the pathological mechanisms of NPSLE.

## Data availability statement

The original contributions presented in the study are included in the article/**Supplementary Material**. Further inquiries can be directed to the corresponding author.

## Ethics statement

The studies involving humans were approved by Biomedical Ethics Committee of the First Affiliated Hospital of Chongqing Medical University. The studies were conducted in accordance with the local legislation and institutional requirements. The participants provided their written informed consent to participate in this study. Written informed consent was obtained from the individual(s) for the publication of any potentially identifiable images or data included in this article.

## Author contributions

WQ: Writing – original draft, Writing – review & editing. SY: Writing – review & editing. LZ: Writing – review & editing. ML: Writing – review & editing, Resources. FL: Writing – review & editing. JT: Methodology, Writing – original draft. JY: Investigation, Writing – original draft. GZ: Conceptualization, Writing – review & editing. XR: Conceptualization, Methodology, Writing – review & editing.
