# Supplementary figures and images for: Kikuchi-Fujimoto disease evolves into lupus encephalopathy characterized by venous sinus thrombosis: a case report
Source: Front Immunol. 2024 Apr 11;15:1389993. doi: 10.3389/fimmu.2024.1389993 (PMC11043565; doi:10.3389/fimmu.2024.1389993)

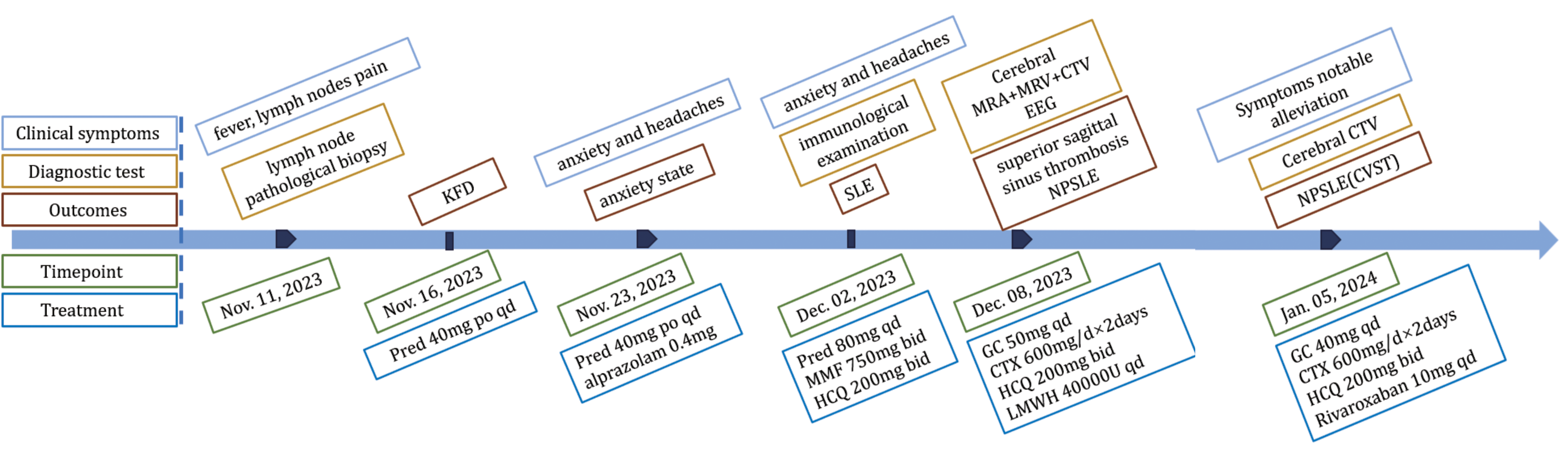

Supplement: Supplementary file 1 [file Image_1.tif]
